# Fulminant Guillain–Barré syndrome secondary to *Campylobacter coli* infection: An autopsy case report

**DOI:** 10.1016/j.ensci.2023.100454

**Published:** 2023-02-25

**Authors:** Fumiya Kutsuna, Momoko Soeda, Aiko Hibino, Masahiro Tokuda, Shiro Miura, Hiroshi Iwanaga

**Affiliations:** a Department of Neurology, National Hospital Organization Nagasaki Medical Center, Kubara 2-1001-1, Omura, Nagasaki 856-0835, Japan; b Department of Pathology, National Hospital Organization Nagasaki Medical Center, Kubara 2-1001-1, Omura, Nagasaki 856-0835, Japan; c Department of Emergency, National Hospital Organization Nagasaki Medical Center, Kubara 2-1001-1, Omura, Nagasaki 856-0835, Japan

**Keywords:** Acute motor sensory axonal neuropathy, Autopsy, *Campylobacter coli*, Fulminant Guillain–Barré syndrome, Immunotherapy

## Abstract

The most common infection preceding Guillain–Barré syndrome (GBS) is *Campylobacter jejuni* enteritis, although a few patients present with *Campylobacter coli*. We report a case of *C. coli*–induced fulminant GBS. A 61-year-old woman presented with bilateral limb weakness. Nerve conduction studies revealed a reduction of amplitude and *C. coli* was isolated from a fecal specimen, leading to the diagnosis of GBS. Although the patient was immediately administered immunoglobulin, her symptoms rapidly worsened and she died. Peripheral nerve autopsy revealed myelin ovoid, and infiltration of CD68-positive macrophages into nerves. More effective treatments for fulminant GBS need to be developed.

## Highlights

• Guillain-Barré syndrome can be rapidly aggravated despite appropriate treatment.
• *Campylobacter coli* might be associated with development of Guillain-Barré syndrome.
• More effective treatments for Guillain-Barré syndrome is needed.

## 1 Introduction

Guillain–Barré syndrome (GBS) is characterized by acute flaccid paralysis, absent or diminished deep tendon reflexes, and autonomic dysfunction. Although the prognosis of GBS is generally considered favorable, approximately 5% of patients die and 20% cannot walk independently at 1 year from onset [1]. GBS remains a serious disease despite treatment with immunotherapies. Severe GBS with rapid clinical deterioration is termed fulminant GBS [2].

The most common infection preceding GBS, especially acute motor axonal neuropathy, is *Campylobacter jejuni* enteritis. The cause of neuronal damage is molecular mimicry between *C. jejuni* lipo-oligosaccharides (LOS) and human gangliosides. Although *Campylobacter coli* also causes enteritis and has similar LOS to *C. jejuni*, its role in GBS development remains controversial. Herein, we report a case of fulminant GBS following *C. coli* infection with a fatal outcome.

## 2 Case report

A 61-year-old woman presented to the emergency department of our hospital with bilateral limb weakness, mild diplopia, and dysarthria that had developed overnight. One week before hospitalization, she had diarrhea and had been diagnosed with enteritis at a local hospital. The patient had a 10-year history of follicular thyroid adenoma, but she had no history of cardiac or respiratory diseases, vaccination, or travel.

At admission, her blood pressure was 179/102 mmHg, pulse rate was 109 beats per minute, and body temperature was 36.9 °C. There were no signs of heart or respiratory failure. Her Glasgow coma scale score was E3V5M6. Physical examination revealed a 7 × 9-cm follicular thyroid adenoma in the left cervical region. Neurological examination revealed muscle weakness (manual muscle testing [MMT] grade 3/5) and absent deep tendon reflexes in all four limbs. Cranial nerve examination revealed oculomotor dysfunction and facial paralysis on the right side. Mild dysarthria and dysphagia were also noted. The results of laboratory test and brain and whole-body computed tomography showed no abnormalities except follicular thyroid adenoma. Nerve conduction studies revealed a reduction of amplitude in the bilateral motor and sensory nerves and reduced F wave frequency, although the nerve conduction velocity was normal, which indicated the axonal degeneration.

Approximately 10 min after admission, the patient rapidly developed respiratory failure and increased muscle weakness in all four limbs (MMT grade 1/5). She was promptly intubated and transferred to the medical intensive care unit. Her medical history, neurological symptoms, and nerve conduction study findings supported the diagnosis of GBS. Therefore, intravenous immunoglobulin therapy at 0.4 g/kg a day was immediately started. On day 2 of admission, her Glasgow coma scale score was E1V1M1 without sedation. Moreover, she developed sinus tachycardia and acute heart failure with a reduced ejection fraction but no ST-segment changes and cardiac enzyme elevation. Although she was administered vasoactive drugs (dopamine, dobutamine, and vasopressin) and used intraaortic balloon pump, she remained hypotensive. On day 3 of admission, she died.

An extensive postmortem examination of autoimmune antibodies revealed positivity for anti-GM1 immunoglobulin (Ig)G and anti-GD1b IgG. No abnormalities in other antibodies, including anti-GQ1b IgG, antinuclear antibody, and onconeural antibodies (anti-amphiphysin, anti-CV2, anti-Ri, anti-Yo, anti-Hu, anti-recoverin, anti-titin, anti-zic4, and anti-GAD65) were noted. *C. coli* was isolated from a fecal specimen.

**Fig. 1:**
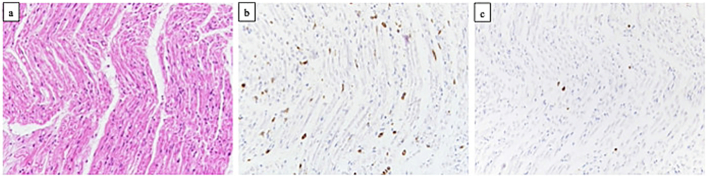
Histopathological findings of peripheral nerve specimens obtained from the brachial plexus bilaterally. Hematoxylin and eosin staining reveals inflammatory cells in the nerve (a). Immunohistochemical staining reveals lymphocytic infiltration of CD68-positive macrophages (CD68 staining) (b) and CD3-positive T lymphocytes (CD3 staining) (c).

An autopsy was performed 12 h after the patient's death. The gross autopsy findings included severe lung edema (total weight: 1305 g) and moderate liver edema (total weight: 1175 g). There were no signs of myocarditis, myocardial infarction, or coronary atherosclerosis. Additionally, we performed microscopic examination of peripheral nerve specimens obtained from the recurrent laryngeal nerve bilaterally and brachial plexus bilaterally with hematoxylin and eosin staining, Klüver–Barrera staining, and immunohistochemical staining (CD68 and CD3 staining) (Fig. 1a-c). A lymphocytic infiltrate was observed in a brachial plexus lesion. The brachial plexus and recurrent laryngeal nerve showed myelin sheath swelling, myelin ovoid, and a reduced number of nerve cells. Furthermore, immunohistochemical staining showed infiltration of CD68-positive macrophages into the nerve. The cause of death was concluded to be cardiogenic shock due to inflammatory polyneuropathy caused by the acute motor sensory axonal neuropathy type of GBS.

## 3 Discussion

We report on a patient with fulminant GBS after *C. coli* infection. She received immunotherapy with immunoglobulin immediately after admission, but she experienced acute clinical deterioration and eventually died. The important clinical issue in this case is whether there are any treatment alternatives for fulminant GBS.

Fulminant GBS is rare and has a more serious prognosis than standard GBS. Despite treatment with immunotherapies, GBS is fatal in approximately 5% of patients [1]. In contrast, the mortality rate of fulminant GBS despite appropriate treatment has been reported to be 14.7% [2]. Therefore, a better treatment is needed. Currently, standard treatments for GBS include intravenous immunoglobulin administration and plasma exchange, with both treatments being equally effective [1]. The advantage of plasma exchange is its rapid effect, and plasma exchange has been suggested to be useful for treating acute progressive GBS. However, plasma exchange can cause large shifts in fluids, and it should be avoided in patients with autonomic instability [1]. Here, plasma exchange was not performed because the patient had autonomic involvement including hypotension and left ventricular dysfunction. Previous studies have estimated the effects of recombinant antibodies that bind to the neonatal Fc receptor for IgG and monoclonal antibodies including eculizumab [1]. These treatments are expected to prevent antibody-mediated nerve injury and promote regeneration. Therefore, these immunotherapies might be more effective for fulminant GBS than standard immunotherapies.

Patients with GBS are generally have flaccid paralysis of extremities, and often develop cranial nerve weakness, usually facial or pharyngeal weakness. On the contrary, opthalmoplegia is rare. In a previous report, 6.5% of patients with GBS had opthalmoplegia [3]. The mechanism of opthalmoplegia of GBS remains controversial, it has been suggested that the relationship between anti-GT1a IgG and opthalmoplegia. Although not confirmed in this case, anti-GT1a IgG may have been related to the symptoms.

Approximately 8.8% of patients with fulminant GBS reportedly have antecedent *C. jejuni* infection [2]. *C. jejuni*–induced GBS typically causes axonal involvement with a slow recovery, which might be related to the severity of GBS. Although *C. jejuni* is the most common cause of campylobacteriosis, *C. coli* can also cause it. Reports of GBS with antecedent *C. coli* infection exist [4], and the other report of *C. coli*-induced GBS has a similar nerve axonal neuropathy to the present case. This may indicate that *C. coli*, like *C. jejuni*, developes GBS due to molecular mimicry. However, the relationship between GBS and *C. coli* infection remains controversial. *C. coli* and *C. jejuni* coexist in the gastrointestinal tracts of animals and enter the environment by contamination with manure. Moreover, co-infection of *C. jejuni* and *C. coli* has been reported in 3.2% of patients with acute flaccid paralysis [4]. Hence, the relationship between GBS and *C. coli* is considered in terms of co-infection with *C. jejuni*. However, the possibility of *C. coli–*induced GBS should not be ignored. A previous report revealed that approximately 0.9% of *C. coli* showed LOS with CstII, which is similar to the LOS of *C. jejuni* and is associated with the development of GBS [5]. Interestingly, horizontal gene transfer between *C. coli* and *C. jejuni* has been reported to occur during adaptation to harsh environments for successful propagation and persistence of species [6]. This mechanism can create hybrid strains, and these new *Campylobacter* spp. might cause GBS. It remains unclear whether *C. coli* plays a role in the development of GBS. Although, this is a single case report and cannot be demonstrated clear results, it is important to consider the relationship between GBS and *Campylobacter* spp. other than *C. jejuni*.

## 4 Conclusion

*C. coli* infection can induce fulminant GBS. Fulminant GBS can rapidly progress despite treatment with immunotherapies. This report emphasizes the need for more effective treatments for GBS, especially fulminant GBS.

## Funding

This research did not receive any specific grant from funding agencies in the public, commercial, or not-for-profit sectors.

## CRediT authorship contribution statement

**Fumiya Kutsuna:** Conceptualization, Writing – original draft, Visualization. **Momoko Soeda:** Resources. **Aiko Hibino:** Visualization, Investigation. **Masahiro Tokuda:** Visualization, Investigation. **Shiro Miura:** Resources. **Hiroshi Iwanaga:** Supervision.

## Declaration of Competing Interest

None.

## Data Availability

Data sharing is not applicable to this article as no new data were created or analyzed in drafting this case report.

## References

[1] Shahrizaila N., Lehmann H.C., Kuwabara S. (2021). Guillain-Barré syndrome. Lancet..

[2] Rougé A., Lemarié J., Gibot S., Bollaert P.E. (2016). Long-term impact after fulminant GBS case report and literature review. Int. Med. Case Rep J..

[3] Bhargava A., Banakar B.F., Pujar G.S., Khichar S. (2014). A study of Guillain-Barré syndrome with reference to cranial neuropathy and its prognostic implication. J. Neurosci. Rural Pract..

[4] Thobela M.S., Smith A.M., Moonsamy S., Plessis D.H., Govender N., Keddy K.H. (2018). Detection of Campylobacter species in stool specimens from patients with symptoms of acute flaccid paralysis in South Africa. J. Infect. Dev. Ctries.

[5] Hull D.M., Harrell E., van Vliet A.H.M., Correa M., Thakur S. (2021). Antimicrobial resistance and interspecies gene transfer in Campylobacter coli and Campylobacter jejuni isolated from food animals, poultry processing, and retail meat in North Carolina, 2018–2019. PLoS One.

[6] Golz J.C., Epping L., Knüver M.T., Borowiak M., Hartkopf F., Deneke C., Malorny B., Semmler T., Stingl K. (2020). Whole genome sequencing reveals extended natural transformation in Campylobacter impacting diagnostics and the pathogens adaptive potential. Sci. Rep..

